# Political Economy of Maternal Child Malnutrition: Experiences about Water, Food, and Nutrition Policies in Pakistan

**DOI:** 10.3390/nu16162642

**Published:** 2024-08-10

**Authors:** Farooq Ahmed, Najma Iqbal Malik, Shamshad Bashir, Nazia Noureen, Jam Bilal Ahmad, Kun Tang

**Affiliations:** 1 Department of Anthropology, The Islamia University of Bahawalpur, Bahawalpur 63100, Pakistan; 2 Department of Psychology, University of Sargodha, Sargodha 40100, Pakistan; najma.iqbal@uos.edu.pk; 3 Department of Psychology, Lahore Garrison University, Lahore 54920, Pakistan; shamshadbashir@lgu.edu.pk; 4 Department of Psychology, Foundation University Rawalpindi Campus, Rawalpindi 58001, Pakistan; nazia.ch88@gmail.com; 5 Taxila Institute of Asian Studies, Quaid-i-Azam University, Islamabad 45320, Pakistan; jam_bilal@hotmail.com; 6 Vanke School of Public Health, Tsinghua University, Beijing 100084, China

**Keywords:** political economics, water insecurity experiences, food insecurity experiences, nutrition policies, social exclusion, mix-methods, Pakistan

## Abstract

This study examined access to water, food, and nutrition programs among marginalized communities in Southern Punjab, Pakistan, and their effects on nutrition. Both qualitative and quantitative data were used in this study. We held two focus group discussions (one with 10 males and one with 10 females) and conducted in-depth interviews with 15 key stakeholders, including 20 mothers and 10 healthcare providers. A survey of 235 households was carried out to evaluate water and food insecurity, with the data analyzed using Wilcoxon’s rank-sum test, *t*-test, and Pearson’s chi-square test. The results revealed that 90% of households experienced moderate-to-severe water insecurity, and 73% faced moderate-to-severe food insecurity. Household water and food insecurity were positively correlated with each other (correlation coefficient = 0.205; *p* = 0.004). Greater household water (*p* = 0.028) and food insecurity (*p* < 0.001) were both associated with higher perceived stress. Furthermore, lower socioeconomic status was strongly related to higher levels of water (*p* < 0.001) and food insecurity (*p* < 0.001). Qualitative findings highlight the impact of colonial and post-colonial policies, which have resulted in water injustice, supply issues, and corruption in water administration. Women face significant challenges in fetching water, including stigma, harassment, and gender vulnerabilities, leading to conflicts and injuries. Water scarcity and poor quality adversely affect sanitation, hygiene, and breastfeeding practices among lactating mothers. Structural adjustment policies have exacerbated inflation and reduced purchasing power. Respondents reported a widespread lack of dietary diversity and food quality. Nutrition programs face obstacles such as the exclusion of people with low social and cultural capital, underfunding, weak monitoring, health sector corruption, and the influence of formula milk companies allied with the medical community and bureaucracy. This study concludes that addressing the macro-political and economic causes of undernutrition should be prioritized to improve nutrition security in Pakistan.

## 1. Introduction

Globally, malnutrition accounts for fifty percent of child mortality cases each year, with Severe Acute Malnutrition (SAM) causing the death of one in every three affected children [1]. The highest rates of underweight and stunting are found in Sub-Saharan Africa and South Asia, with 78% of wasted children residing in Bangladesh, India, and Pakistan [2,3,4,5]. However, Pakistan has a higher ratio of child malnutrition cases compared to its South Asian counterparts [6,7]. The Pakistan Demographic and Health Survey 2018 indicates that mortality is highest among the most deprived sections of the population [8]. Under-nutrition among children under five is more prevalent in the poorest and most rural areas compared to wealthiest quintiles and urban regions [9]. In rural areas, 44% of children suffer from stunting, 32% are underweight, and 19% experience wasting. In contrast, urban areas report lower rates: 35% stunting, 24% underweight, and 17% wasting [9]. Southern Pakistan regions, such as Sindh, Baluchistan, and South Punjab, have stunting rates between 45–48%, higher than the KP, ICT, and national averages of 35–40%. Within Punjab, South Punjab shows a significantly higher rate of underweight children (26.4%) compared to the rest of Punjab (18.7%), with the highest prevalence in the districts of Rajanpur (34%), Rahim Yar Khan (33%), and D.G. Khan (32%) [10].

Recent systematic reviews in developing countries reveal that multiple social, economic, and political factors indirectly or directly influence the nutritional status of children [11], for example, antenatal care [12], higher fertility [13], maternal illiteracy [14], maternal stress, breastfeeding frequency [15], low caste or class [16], poverty [17], lack of food intake owing to a decline in production [18], and area of residence [19]. These factors can act separately or in combination. In addition, the connection between water, food, and nutrition is well recognized [20]. Water is essential for maintaining a healthy environment. Effective water management is crucial for mitigating water and food insecurity at the household level [21]. Furthermore, effective public nutrition programs are crucial for reducing malnutrition rates [22]. In Pakistan, development programs have been unsuccessful in addressing malnutrition because they have overlooked the entrenched social structures of inequality. The Benazir Income Support Program (BISP), proposed by the World Bank and the world’s largest social protection initiative in Pakistan post-2010, proved less effective in improving the status of poor women, ensuring food security, and alleviating hunger due to its disregard for socio-cultural power dynamics. Also, the Community Management of Acute Malnutrition (CMAM), a temporary therapeutic program targeting SAM, was implemented in impoverished southern districts; however, these interventions failed to consider ongoing power dynamics and ignored unequal social relationships [23].

This study contends that malnutrition stems from unequal access to various essential resources. People’s access to fundamental nutritional resources, market commodities, and development programs is influenced by their social, economic, and cultural capital. Social groups from remote, rural areas and those belonging to lower castes, classes, and capital are often deprived [24]. Critical medical anthropologists assert that development and biomedical approaches typically focus on curing the “individual body” rather than addressing the “social body”. They argue that these approaches fail to consider humans and their experiences of insecurity, illness, and suffering from a holistic perspective [25]. Political economics is essential for understanding the formulation and implementation of public policies and their broader impacts on the economy and social systems. Prominent programs and policies such as the “Green Revolution” and “Structural Adjustments”, which shaped the lives of people in developing countries, underscore the significant influence of neoliberal hegemony [26,27].

Politico-economic factors such as colonial history, political instability, structural adjustments, privatization, inadequate market regulation, and foreign debt policies have led to a lack of comprehensive social welfare and unmet governance promises [28,29,30,31]. As a result, the state has struggled to manage inflation and provide equitable health and nutrition services. In their most critical periods, mothers and children require diverse social determinants for their well-being [32]. However, in developing countries such as Pakistan, low trade, a fragile economy, and corruption hinder the provision of quality services. Consequently, various groups, including those in the southern regions, face marginalized and insecure living conditions due to their limited access to resources in a privatized, market-driven economy. Previous public health research on malnutrition in Pakistan has often overlooked these broader structural issues, instead attributing problems to local cultures under the influence of neoliberalism [23].

Malnutrition rates are highest in Africa and South Asia, regions that were once colonized. In Pakistan, malnutrition can be seen as a significant postcolonial and post-development challenge. The social, cultural, and economic engineering during colonial and postcolonial periods contributed to the current high prevalence of malnutrition in low-priority areas [25,26]. Resource control was designed and managed to replace indigenous knowledge and practices, reinforce hierarchical inequalities, and foster dependency on external assistance. Interventions to combat malnutrition have often disregarded local socio-cultural contexts, leading to failures in providing equitable access to essential resources.

The impact of political economy has seldom been considered in understanding how colonial, neo-colonial, and neoliberal influences and regional disparities are linked to structural vulnerability. To address this gap, the present study deconstructs the politico-economic factors that adversely affect potential nutritional resources, including water, food, and community-level nutrition programs. This study aims to investigate how experiences of resource insecurity contribute to malnutrition in Southern Pakistan. We hypothesized that household water insecurity is positively correlated with household food insecurity. We further hypothesize that households’ lower socioeconomic status and higher perceived stress levels are strongly associated with increased household water and food insecurity.

## 2. Materials and Methods

### 2.1. Data Collection and Analysis

**Figure 1:**
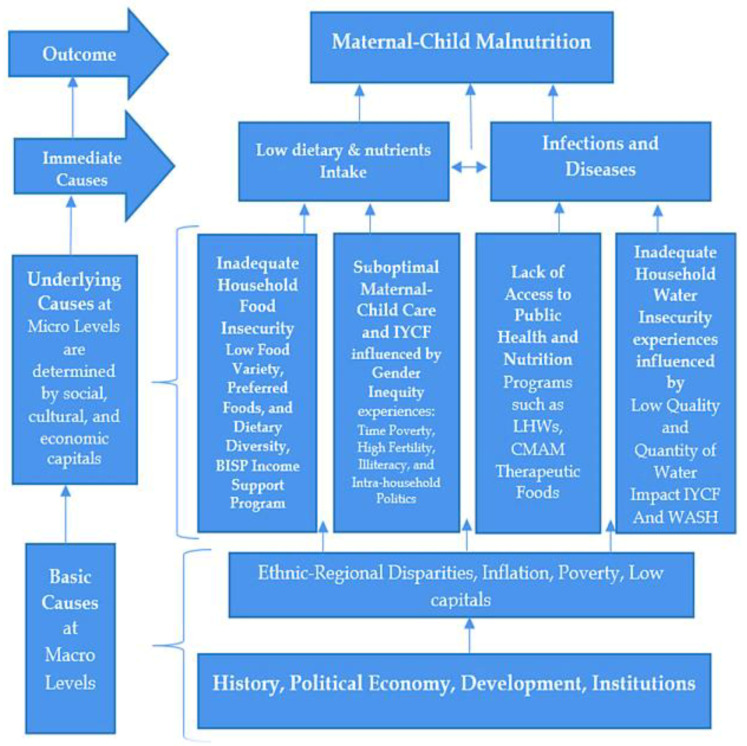
Conceptual framework showing causes of malnutrition.

The conceptual framework developed by UNICEF (1990) showed mortality and malnutrition as a collective outcome of immediate, underlying, and basic causes. The immediate causes (illness) depend on the underlying causes (inadequate access to food, care, and a healthy environment). Inadequate access is, however, shaped by the following basic causes: political, economic, institutional, ideologies, and policies. The conceptual framework provided below has been adjusted to fit the local context and specific needs. We explore, examine, and interpret experiences about the lack of access to potential resources, particularly water security, food security, public health nutrition programs, and care. We describe how the lack of social and cultural capital affects access to nutritional-specific and sensitive programs controlled by power institutions such as bureaucracy (Figure 1).

Critical medical-nutritional anthropologists frequently suggest examining the political and economic context of local food and water insecurity. This involves conducting in-depth interviews alongside household surveys to better understand and address the nuances of these issues [33]. Therefore, for this study, both qualitative and quantitative methods were employed. First, using snowball sampling, we gathered data from five key informants who had profound knowledge about the area, history, and geography. With the help of these key informants, two focus group discussions (one with males and one with females) were conducted in the most water-insecure areas. In each focus group, which lasted nearly two hours, a maximum of 10 participants were allowed to participate. The group discussions and key informant interviews covered topics such as water justice and the coping strategies of marginalized communities, issues related to water fetching and gender sensitivities, and impacts on agriculture, livestock, Water, Sanitation, and Hygiene (WASH), and Infant and Young Child Feeding (IYCF). Questions also addressed food insecurity, focusing on food affordability, quality versus quantity, and food diversity. 

Next, using purposive sampling methods, we started in-depth interviews with key stakeholders, focusing first on healthcare providers (supply side) and then on the mothers of malnourished children (demand side) who were registered and enrolled in the therapeutic program. As interviews were conducted first with healthcare providers, nutrition experts, and key officials from the Health Department (*n* = 10), it helped identify 30 mothers of severely malnourished children. However, we could obtain consent from only 20 mothers after navigating the gatekeepers (Table 1).

An interview guide was created to explore the significant structural challenges and barriers faced by women during treatment and therapeutic coverage. This guide was pilot-tested with a few mothers. To ensure comfort and engagement, interviews were conducted at the local residences of the participants. Mothers were queried about their access to healthcare, nutrition-specific and sensitive programs, nutrition stabilization centers, and lady health workers, as well as the obstacles and challenges they face in gaining access to these services. Face-to-face semi-structured interviews were conducted in the participants’ local Seraiki language, allowing for a flexible format that lasted between 1–2 h. The sociodemographic characteristics of these mother are given in Table 2.

**Figure 2:**
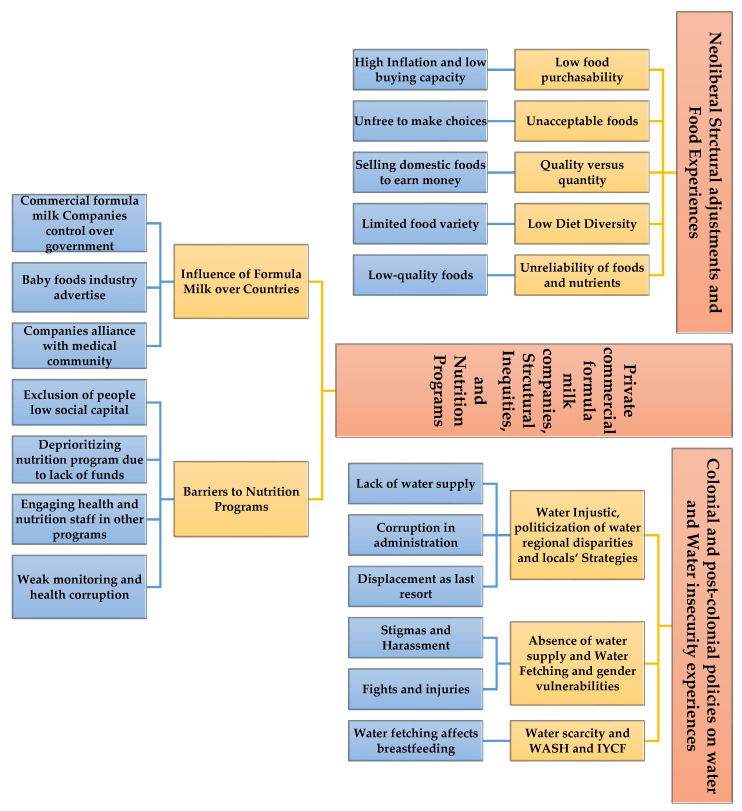
Graphic abstract showing insecurity experiences about water, food, and nutrition programs.

All raw qualitative data and field notes gathered during focus groups, KII, and IDI were carefully reviewed and translated verbatim into English. Key texts and narratives were coded and organized based on common meanings and categories by two researchers. Then, we congregated similar codes to produce wider classifications. Next, narratives were authenticated, inconsistencies were removed, categories were reassessed, and frequently visible subthemes began evolving from the whole data. Both inductive and deductive methods were used by these researchers to identify potential themes and sub-themes from the qualitative data analysis. In the end, three major themes and multiple sub-themes emerged from qualitative data, which have been arranged in three parts in the diagram given below (Figure 2).

Secondly, this study utilized data from a small-scale survey (*n* = 235) conducted in 2018 in water-insecure districts of South Punjab to assess experiences related to water and food insecurity. The FGDs we mentioned earlier were instrumental in determining the survey areas. Additionally, the individuals involved in both the FGDs and the survey were from the same group and communities. The first author (F.A.), a co-investigator from Pakistan and collaborator with an international water consortium, helped develop the Household Water InSecurity Experiences (HWISE) Scale. This scale, used across various cultural settings in 23 low- and middle-income countries, assessed household water insecurity through 34 items covering water attainment, usage, and storage, as well as food insecurity. After applying item-response theories and classical-test methods, 12 items were retained for analysis. Factor analyses demonstrated that these items were unidimensional and reliable (Cronbach’s alpha 0.832), and the scale established construct, predictive, convergent, and discriminant validity [34]. The questionnaire was translated from English into Urdu and Seraiki by the first author and adapted for use in South Punjab. 

Based on previous area information and key informants’ knowledge, households from water insecure clusters were selected through cluster sampling (selection of insecure clusters) and Simple Random Sampling (WHO walk every 3rd HHD) techniques. Enumerators, selected for their experience in survey implementation, familiarity with the area, and fluency in local languages, underwent a 1- to 2-day training. Each day, at the end of the interviews, these surveyors were debriefed, and detailed field notes were extracted. In addition to the items on water insecurity described earlier, data were collected on socio-demographic characteristics, household food insecurity using the Household Food Insecurity Access Scale, and perceived stress using a modified 4-item perceived stress scale.

**Figure 3:**
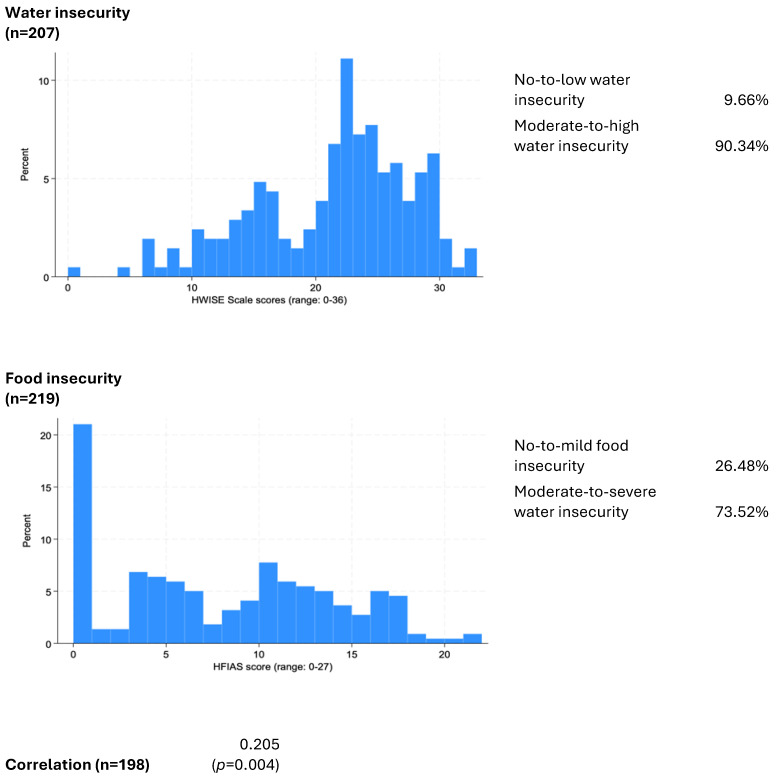
Correlation between continuous food and water insecurity scores.

Household Food Insecurity Access Scale (HFIAS) scores and associated indicators, as well as Household Water Insecurity Experiences (HWISE) Scale scores and associated indicators, were calculated using a standardized methodology. Briefly, responses (0 = “never”, 1 = “rarely”, 2 = “sometimes”, 3 = “often” or “always”) to each of the 12 items were summed [34]. For all items, responses of “do not know” or “not applicable” were coded as missing, except for the question about water interruptions, for which “not applicable” was recoded as zero. Households were classified as having no-to-marginal (HWISE Scale scores of 0–2), low (3–11), moderate (12–23), or high (24–36). Basic descriptive statistics (Wilcoxon’s rank-sum test, *t*-test, and Pearson’s chi-square test) were used to assess whether sociodemographic characteristics, perceived stress, and experiences with resource insecurity varied by water or food insecurity status. We also examined whether continuous food and water insecurity scores were correlated with each other (see Figure 3). Analyses were completed using Stata (v18).

Of a total of 235, 42.55% were male and 57.45% were female. The majority belonged to the occupations of agriculture (<50%) and labor (~22%). The primary monthly income of the majority of participants was less than RS. 20,000 PKR (<100 USD), see Table 3.

### 2.2. Ethical Consideration

The Advanced Studies and Research Board of Quaid-i-Azam University Islamabad provided approval for this study. Along with obtaining oral consent from the local population, the purpose and nature of this research were communicated to the participants. In addition, this research strictly adheres to the well-versed approval, confidentiality, privacy, and anonymity of all the study respondents. To seek their formal consent, we informed all these mothers about the kind of research. Respondents’ places were deliberately chosen so they could not feel uncomfortable during probing. We also did not use audio recorders due to the cultural sensitivity of local norms.

### 2.3. Strength and Limitation

The methodology has some limitations. First, translating questionnaires from English to the local language and then back to English may have impacted the accuracy of the meanings. Second, a few respondents showed feelings of mistrust or evasion regarding the survey. Additionally, conducting two separate investigations—focus groups and the survey—in the same community at different times presents a methodological limitation. Nevertheless, using qualitative methods alongside the structured survey offers triangulation, which strengthens this study by validating the results.

## 3. Results

### 3.1. Quantitative Water and Food Insecurity Experiences

The hypothesis that a positive correlation exists between continuous food and water insecurity scores was confirmed by the statistical analysis, with a *p*-value of 0.004, indicating statistical significance between HWISE and HFIAS scores (Figure 3).

The interpretation of our results in Table 4 is as follows: (1) greater number of children in the household associated with greater water insecurity; (2) lower monthly income associated with greater water insecurity (*p* < 0.001); (3) lower perceived SES associated with greater water insecurity (*p* < 0.001); (4) greater access to a basic drinking water source (relative to a less-than-basic water source) associated with greater water insecurity; (5) drinking water thought to be unsafe in the prior month associated with greater water insecurity; (6) borrowing water from others in the prior month associated with greater water insecurity (*p* < 0.001); (7) higher perceived stress associated with greater water insecurity (*p* = 0.028); (8) greater percentage of households with high water insecurity experience severe food insecurity relative to households with no-to-low or moderate water insecurity.

The interpretation of our results in Table 5 is as follows: (1) households headed by women are less likely to have severe food insecurity; (2) lower monthly income is associated with greater food insecurity (*p* < 0.001); (3) lower perceived SES is associated with greater food insecurity (*p* < 0.001); and (4) higher perceived stress is associated with greater food insecurity (*p* < 0.001).

Our other hypotheses that lower socioeconomic status and high stress are associated with greater water insecurity and food insecurity are established by the statistical analysis, with the *p*-values demonstrating positive statistical significance as shown in Table 4 and Table 5.

### 3.2. Qualitative Water and Food Insecurity Experiences

Inadequate, unsafe, and low-quality water and food, combined with limited access to healthcare and nutrition programs, negatively impact community health and nutrition. Marginalized communities’ experiences with these vital resources need to be explored at deeper levels to link with the high malnutrition prevalence in southern regions.

#### 3.2.1. Water Insecurity Experiences at the Community Level

Water security is an essential component of human health and nutrition. Water insecurity experiences at the community level can help us understand the major causes of the problem and the associated implications for the health of mothers and children. The following major themes and sub-themes emerged from qualitative data (Table 6).

#### 3.2.2. Food Insecurity Experiences at Community Level

Food insecurity and malnutrition are inseparable. Understanding our experiences with food insecurity can help us determine the underlying causes of maternal-child malnutrition. The following major themes and subthemes emerged from qualitative data (Table 7).

#### 3.2.3. Experiences with Nutritional Programs and Policies

Nutrition-specific and sensitive programs are supposed to facilitate malnourished mothers and children. Poor and marginalized mothers’ experiences of negotiating these programs can better explain the real situation on the ground. Table 8 explores the structural barriers, inequities, and governmentality mothers face at the community level.

## 4. Discussion

This study examined whether continuous food and water insecurity scores were correlated with each other and assessed whether sociodemographic characteristics, perceived stress, and experiences with resource insecurity varied by water or food insecurity status. The quantitative data analysis validated the first hypothesis, showing a positive correlation between household water insecurity and food insecurity with a significant *p*-value of 0.004 (Figure 3). Additionally, lower socioeconomic status was linked to higher levels (*p* < 0.001) of both water and food insecurity (Table 4 and Table 5 respectively). The hypothesis that higher perceived stress is associated with increased household water and food insecurity was also confirmed, with statistically significant *p*-values (*p* = 0.028 for water insecurity and *p* < 0.001 for food insecurity). In sub-Saharan Africa, significant links between resource insecurity and psychological health have been observed, with research highlighting strong correlations between stress and limited access to preferred food and water [35]. These results are consistent with recent studies in low- and middle-income settings, which attribute water and food insecurity to structural causes at lower levels. These insecurities are interrelated and contribute to a syndemic relationship with stress and overall well-being [36,37]. 

The findings indicated that water injustice, by interacting with food insecurity, inadequate WASH and IYCF practices, maternal illness, and low breastfeeding, contributes to both acute and chronic malnutrition at the microlevel. Low-income households have faced reduced dietary diversity and a limited range of foods due to ongoing poverty and inflation. It is generally accepted that economic poverty causes mental distress, but a shortage of food or water can also induce stress [38]. The distress caused by water and food insecurity may be complex, as both are significant contributing factors [39]. Stress can arise from various mechanisms, such as material deprivation, shame or stigma, concerns about health and safety, interpersonal conflict, intimate partner violence, and institutional injustice [40]. Several studies emphasize the links between food and water insecurity, highlighting the importance of advocating for policies and interventions that simultaneously address all resource insecurity issues by governmental and non-governmental organizations [41,42,43]. 

### 4.1. Political Economy of Water Insecurity

Qualitative data reveal that water insecurity in southern Punjab is primarily due to water injustice. Specifically, canal water in this region is available for less than six months a year, unlike the rest of Punjab, where it is accessible year-round. This water insecurity affects maternal and child health through various pathways, including gender inequities and inadequate IYCF and WASH conditions. The southern areas are particularly disadvantaged due to the macro-level water situation. Evidence indicates that micro-level water distribution has been consistently neglected. The current irrigation system, which relies on barrages, weirs, and permanent headworks established during British colonial rule, replaced the traditional system of seasonal canals and disrupted the local harvesting practices that were well-suited to the environment [44]. The primary motivation behind this shift in irrigation in central and southwest Punjab was to develop canal colonies and clusters, creating new socio-cultural and economic structures [45]. During the colonial period from 1885 to 1947, over a million people were relocated to nine canal settlements. This led to the creation of a gravity-based, multi-level canal system, which ultimately resulted in a hierarchically controlled network of sub-canals, disadvantaging tail-end farmers due to issues with village course inlets or the Moga system [46] (p. 30), which is consistent with our findings.

Instead of directly addressing individual or farmer access to water, the distribution was influenced by another irrigation control factor: the amount of land connected to each outlet [47]. Under British rule, land proportions became a tool for water allocation, benefiting landlords and creating a discriminatory land division that hindered fair water distribution in Punjab [48]. Colonial policies reinforced the power of rural elites, perpetuated social stratification and class manipulation, and prevented lower sections of society from fully benefiting from agriculture [49]. The undue advantage given to large landholders exacerbated livelihood insecurity as the concurrent mechanization of agriculture continued to shape the social reality of water access [46].

The Green Revolution primarily benefited large landholders who could afford the costly new machinery, leaving small landholders unable to take advantage of the agricultural advancements. Consequently, between 1960 and 1990, tenant farms decreased by nearly fifty percent, while the rural population without land—working as daily wage laborers—increased by forty percent, leading to heightened livelihood insecurity and urban migration [50]. Additionally, the Punjab Irrigation Department, acting as the sole mediator without police or judicial oversight, often failed to address issues with the Moga system and struggled to ensure fair distribution due to inadequate auditing and checks and balances. Favoritism and nepotism allowed big landlords to secure a disproportionate share of water, resulting in frequent protests by small farmers and peasants. The revenue and water tax (Abiana) are contentious, and the enforcement of agricultural labor laws is problematic. The lack of accountability and the impunity of powerful landlords cannot be addressed in isolation but require reforms in bureaucracy and politics [46]. 

### 4.2. Political Economy of Food Insecurity

Qualitative data analysis reveals that while extreme hunger is not widespread among households, most respondents are dissatisfied with the diversity and affordability of their food, with wheat being the primary staple. Research shows that neoliberal policies have been central to Pakistan’s economy. The “Green Revolution” in the 1960s, supported by USAID, mainly benefited landlords rather than peasants. Cash crops such as sugar, wheat, and cotton use more water and dangerous pesticides, ultimately reducing food diversity. By the eighties, Pakistan, once self-sufficient in wheat and rice production, became food-dependent again due to global cuts in food prices, leading to reduced grain demand and a formal decline in the agriculture sector [51].

Although imports increased to 2.5 million tons a decade later, hunger and malnutrition persisted due to unfair resource distribution and weak purchasing power. In 2007, as global food grain prices rose, Pakistan was forced to keep exporting its limited wheat surplus under international financial pressures, extending subsistence challenges. Foreign countries and agribusiness investors began acquiring agricultural lands on long-term leases, with all grains intended for export back to the investing countries [52].

Efforts to combat hunger and malnutrition were undermined by minimal state intervention under neoliberal economic policies, keeping food prices high. Wheat prices rose from PKR 400 to PKR 630 per 40 kg between 2004 and 2008, reaching PKR 1400 per 40 kg in 2019 and PKR 2200 per 40 kg in 2022 [53]. Increased international demand led to wheat smuggling, exacerbating the crisis. Additionally, rising petroleum, gas, and fertilizer prices impacted agricultural production, forcing the government to import wheat at high costs of USD 300 per ton. The consumer price index rose by 25%, and wheat prices increased by 20% [54]. The decline in agricultural investment from 2.1% to 1.1% (1999–2009), low-quality research, poor monitoring and evaluation, weak federal policy coordination, and poor provincial implementation intensified hunger and malnutrition [54].

Policy reform was never seriously undertaken but adhered to the demands of the World Bank and the Asian Development Bank for balance of payment and budgetary support. A comprehensive, long-term approach to agricultural development and poverty reduction is impossible without good governance and political reform. Policymakers’ focus on production has undermined the accessibility issue for the poor [51]. Addressing issues such as storage, transportation, irrigation, and agriculture are secondary measures; unequal land distribution is the primary structural issue, as access to land would reduce relative food prices [51]. The World Bank [55] found that from 2000 to 2014, food costs rose by 270%, while non-food item prices increased by 180%. Household-level food security is not supported by cash crops such as cotton and sugarcane [56]. In South Punjab, maternal nutrition issues remain unaddressed due to strict household food budgets, intra-household food distribution, economic decline [50], and gender and social inequities. From 1990 to 2008, the number of malnourished individuals rose from 24 to 45 million nationwide. The prices of key items, from staple crops to cooking oil, increased by almost 20% in early 2010, with uncertainty expected to continue, as indicated by the wholesale price index [48]. In May 2022, food prices surged by over 50%, further weakening the already fragile purchasing power of the poor.

### 4.3. Political-Economy of Nutrition Programs

Our findings indicated a temporary and unsustainable nutrition policy. It corroborated the historical evidence that shows inconsistent national strategies, including food distribution, card-based rationing, wheat subsidies, and distribution to flood victims in Punjab, along with recent federal cash transfer programs for poor women through BISP [57]. UN agencies (UNICEF, WFP, FAO, and WHO) have led various nutritional programs involving health departments in the past. Evidence shows that the formula milk industry often ignored laws and continued advertising their products unlawfully [58]. This illegal and unethical promotion increased malnutrition by depriving children of their mother’s milk. Reports revealed that 80% of mothers were instructed by healthcare providers to give formula milk [59]. In theory only, the Punjab Food Authority (PFA) has had 16 laws approved by the Punjab Assembly, banning the marketing and sampling of infant formula milk in hospitals after consulting with the Formula Milk Association. Authorization for formula milk ingredients and marketing is now required from the PFA scientific panel, and all imported products must be labeled in the national language [60].

The targeting of beneficiaries for cash transfers was flawed, as evidence showed that deserving widows and domestic female servants were excluded [61]. Power dynamics control development and poverty alleviation programs [62,63]. Connections to local politicians are crucial for becoming BISP beneficiaries in Pakistan [61]. Deserving families face not only social-structural hurdles but also technicalities and complexities related to the selection process. Therefore, factors leading to social exclusion should be central to advising on program objectives, beneficiary selection, and nomination [62]. On Pakistani bureaucracy, Hull [63] concluded that “A powerful person can move a ‘stuck up’ file. Those without influence have to ‘put wheels on it. Money or political influence can affect not only the speed but the path as well, diverting a file from its normal trajectory”. Although the program aimed to uplift the poor through cash grants of PKR 5000 per month to millions of households, reports indicate previous governments misused taxpayers’ money (an annual budget of PKR 100 billion for 5.2 million of the poorest women) for political supporters, depriving genuinely deserving individuals. Profiling of BISP beneficiaries revealed that 1.42 million beneficiaries were government employees, including 2543 in grades 17 to 21; 153,302 had taken overseas trips once; 10,476 traveled more than once; 692 were vehicle owners; and 43,746 spouses owned one or more cars [64].

CMAM is also a temporary remedial strategy, particularly in crisis settings. Yet, the sustainable solution to maternal and child undernutrition lies in the social and economic empowerment and education of women [65]. Empirical data shows that the multisectoral solution strategy seems less effective; thus, social safety nets for poor females necessitate micro-level nutrition-sensitive and nutrition-specific interventions, especially after devolution [56]. However, remote areas remain uncovered, and the government often ignores them except for the better-off, indicating a significant gap between theory and practice. Detached from local socio-cultural realities, the global technical solution under RUTFs and CMAM was implemented “under neoliberal governments and facilitated an increasingly inequitable economy with minimal state involvement in an increasingly individualistic social environment” [65] (p. 16). Therefore, forming the Multi-Sectoral Nutrition Centre (MSNC) in the Planning and Development Department at the provincial level is not a genuine solution.

Findings showed that malnutrition has particularly affected women and children from remote areas, especially those who are water-insecure, unemployed, low-income, daily wage workers, domestic household servants, lower caste individuals, illiterate, and poor mothers with high fertility and work burdens. This suggests that ethnic-regional inequalities at the provincial level, the rural-urban gap, and caste-class stratification at the micro level must be addressed through concrete measures [23,66,67,68]. Ineffective coverage and managerial inefficiencies are evident, with up to 50% of the population in several rural districts not covered by LHWs, especially in the most remote and poorest areas [69]. This research underscores how overarching political and economic factors—such as neoliberal programs, post-colonial bureaucracy, and regional inequalities—diminish essential resources for southern regions. This creates a harmful cycle of inequality that leads to structural vulnerabilities.

## 5. Conclusions

This study confirms that water and food insecurity are interconnected, with increased levels of both insecurities linked to lower socioeconomic status and higher perceived stress. Community experiences with water insecurity, low food diversity, structural challenges to therapeutic coverage, and the unrestricted use of formula milk products suggest that Pakistan has been significantly influenced by colonial and neoliberal policies. Government practices and policies have promoted privatization and biomedical solutions rather than addressing social stratification. Instead of tackling local problems with tailored solutions, the state has relied on global remedies. Policymakers can take specific actions to address these challenges. Ensuring water justice in southern Pakistan is crucial. Implementing land reforms and efficiently using water resources will help alleviate food insecurity. Nutrition-specific and sensitive programs (both therapeutic and social safety nets) must address the underlying issues of social inclusion, caste and class structures, rural-urban disparities, and structural vulnerabilities. Combating corruption and management issues is an important aspect and requires the rule of law and good governance at the macro level.

## Figures and Tables

**Table 1:** Details about the Respondents.

Details about Discussion and Interviews of This Study	No of Respondents (n)
2 FGDs (1 with males and 1 with Females)	20
Key Informant Interviews in the Community	5
Key Informant Interviews with Healthcare Providers	5
Key Informant Interviews Officers in Nutrition Stabilization Centers	5
In-depth Interviews of local mothers availing Nutrition Programs	20

**Table 2:** Characteristics of Mothers (*n* = 20).

Indicator	Frequency	Percentage
**Mothers’ Age**		
18 to 24	6	(30%)
25 to 29	5	(25%)
30 to 34	5	(25%)
34 to 40	4	(20%)
**Literacy of Mothers**		
Illiterate	16	(80%)
~5th–8th	3	(15%)
~10th	1	(5%)
15 to 20	2	(10%)
**Occupation of Mothers**		
Agriculture	11	(55%)
Domestic labour	7	(35%)
Other	2	(10%)
**Income of Household per Month**		
~10 K PKR (~90 USD)	10	(50%)
~15 K PKR (~135 USD)	7	(35%)
≥16 K PKR (~150 USD)	3	(15%)

**Table 3:** Sociodemographic Characteristics of Participants (*n* = 235).

Indicator	Frequency	Percentage
**Sex of Participants**		
Female	135	57.45%
Male	100	42.55%
**Occupation of Household Head**		
Cultivation	110	46.80%
Laborers	52	22.12%
Small business	30	12.76%
Basic subsistence	28	11.91%
Salaried	13	5.53%
**Monthly Income of Household**		
≤Rs. 10,000 (~90 USD)	135	57.45%
≤Rs. 20,000 (~180 USD)	56	23.82%
≥Rs. 21,000 (~200 USD)	44	18.72%

Source: Household Water and Food Insecurity Survey 2018.

**Table 4:** Household water characteristics in South Punjab, Pakistan (*n* = 207).

	No-to-Low Water Insecurity	Moderate Water Insecurity	High Water Insecurity	
	(N = 20)	(N = 108)	(N = 79)	*p*
**Woman household head**	5.0%	10.2%	11.4%	0.699
**Respondent age (years),** mean ± SD	35.3 ± 8.5	34.3 ± 9.6	36.0 ± 9.7	0.471
**Number of children living in household,** mean ± SD	3.0 ± 1.8	3.6 ± 2.0	4.8 ± 2.2	<0.001
**Monthly income (USD),** median (IQR)	99 (47–180)	90 (59–180)	63 (45–99)	<0.001
**Perceived SES standing (1 = best, 10 = worst),** mean ± SD	6.3 ± 1.5	7.3 ± 1.5	8.0 ± 1.1	<0.001
**Basic drinking water source**	73.7%	92.4%	94.9%	0.010
**Drank water thought to be unsafe**	80.0%	98.1%	100.0%	<0.001
**Borrowed water from others**	57.9%	84.8%	96.2%	<0.001
**Perceived Stress Score (range: 0–16),** median (IQR)	4 (2–5)	5 (4–9)	7 (3–10)	0.028
**HFIAS score (range: 0–27),** median (IQR)	5 (0–9)	7 (3–12)	8 (0–14)	0.201
**Food insecurity category**				
No-to-mild	33.3%	23.5%	30.8%	0.041
Moderate	38.9%	43.1%	21.8%	
Severe	27.8%	33.3%	47.4%	

HWISE Data.

**Table 5:** Household food characteristics in South Punjab, Pakistan (*n* = 219).

	No-to-Mild Food Insecurity	Moderate Food Insecurity	Severe Food Insecurity	
	(N = 58)	(N = 75)	(N = 86)	*p*
**Woman household head**	17.2%	5.3%	7.0%	0.041
**Respondent age (years),** mean ± SD	33.7 ± 8.5	35.7 ± 9.7	36.3 ± 10.1	0.268
**Number of children living in household,** mean ± SD	4.1 ± 2.3	3.5 ± 2.0	4.3 ± 2.2	0.065
**Monthly income (USD),** median (IQR)	144 (90–225)	108 (63–180)	63 (45–90)	<0.001
**Perceived SES standing (1 = best, 10 = worst),** mean ± SD	6.9 ± 1.6	6.8 ± 1.7	8.3 ± 1.0	<0.001
**Perceived Stress Score (range: 0–16),** median (IQR)	4 (2–4)	4 (4–9)	8 (6–10)	<0.001

HWISE Data.

**Table 6:** Water Insecurity Experiences.

Theme	Sub-Theme	Narratives
Water injustice and communities’ coping strategies	Absence of water supply and availability of bad-quality water	“In the past, water distribution was much better, but now it primarily benefits large landlords and people in power. Small landholders in the South frequently experience water shortages. This change began after colonization and land control, and the situation worsened when landlords started profiting from cash crops in the 1960s”. (KII, Male, 48) “Canal water distribution in the South Punjab region is unfair, as water is available for less than six months. The canals are controlled by bureaucracy. In many areas of the D.G. Khan division, floodwater is collected in ditches because the underground water is heavy and salty. There is no water supply available here, so water supply schemes are essential. People rely solely on rain or floodwater and pray for rain in the Suleiman Mountains. The responsibility of carrying water primarily falls on women and children”. (KII, Male, 45) “The public water supply is consistently unreliable, and the available water is unclean. We have no choice but to use this poor-quality water. The government supports foreign private companies in selling water, but we can’t afford bottled water, so we are forced to drink the unclean water”. (FGD, Mother, 34)
	Corruption in administration	“The canal’s width is narrow, and powerful individuals illegally divert water by creating cuts due to corruption in the irrigation department. As a result, the water level at the tail end is reduced, leaving insufficient water for crops”. (KII, Male, 57)
	Displacement as a last resort	“People often have to migrate when the water supply runs out. During their journey, they frequently become homeless and lack access to food, water, and toilets”. (FGD, Male, 53)
Water fetching and gender vulnerabilities	Stigmas and harassment	“People may provide water, but they demand something difficult in return. Harassment and even rape are common occurrences while fetching water. (KII, Female, 53)
	Fetching water difficulties	“Fetching water is exhausting; it takes children and women an hour, and in the summer, it becomes even greater challenge”. (FGD, Mother, 27)
	Fights and injuries	“Fetching water results in health problems, injuries, and conflicts”.
Water scarcity, WASH and IYCF	Feeding requires safe water	“Dirty and muddy water often makes our young children sick and contaminates our food. Doctors recommend mineral water from private companies for sick children, but it is too expensive for most poor and rural mothers to afford”. (FGD, Female, 26)
	Fetching affects breastfeeding behaviors	“During the summer months of June, July, and August, the water situation causes significant stress for mothers, leading to increased maternal stress. Consequently, infants suffer due to reduced breastfeeding”. (FGD, Female, 19)
Water-food nexus	Low agricultural production	“We can’t grow crops during water shortages, which causes our lands to dry up. As hunger increases, we are forced to sell our land at low prices and migrate to earn money for survival”. (KII, Female, 53)
	Less milk production	“Our cattle have stopped producing milk due to a lack of food. When our livestock drink less water, their milk production decreases significantly”. (FGD, Male, 40)

Source: Field data.

**Table 7:** Food Insecurity Experiences.

Themes	Sub-Themes	Narratives
Diet quality vs. quantity	Daily diet or staple food	“The government historically supported profitable crops like tobacco, sugar, cotton, and wheat, which significantly reduced the cultivation of fruits and vegetables”. (KII, 45) “While a variety of items are available in the market, wheat remains the staple diet for most people here. The poor mainly eat wheat bread with a mixture of mint, green chili, and onion”. (KII, 38)
	Inflation reduces buying capacity	“Inflation has made our lives very difficult; we dilute a liter of milk with water to stretch its quantity. Meat and fruit are rare in our diet because they are too expensive. Everyone seems worried and mentally stressed due to the rampant inflation”. (FGD, Mother, 34)
Preferred vs. disliked food	Unable to make choices freely	“Highly marginalized household domestic workers often collect expired or leftover food from the homes where they work. To manage the smell, we heat the food because we can’t afford to buy fresh items”. (IDI, Domestic household servant, 29)
Food availability and accessibility	Selling domestic food items to earn a little money	“Poor rural people often sell milk, eggs, or chickens in the local market to earn a little money, but their children often go hungry. They are compelled to sell these items, especially when they are ill or need money for medical treatment. One day at the market, I saw two young children selling a chicken. I asked how much they were selling it for, and the older boy said ‘400 rupees.’ After I paid and took the chicken, the younger child began to cry. I asked him why he was crying, and his older brother said, ‘There is nothing.’ I was puzzled and asked the older brother to explain. The older boy tearfully revealed that the chicken belonged to his younger brother, who had also eaten its eggs. They were selling it out of necessity because their mother was very sick, and they needed the money for her treatment. The younger brother was distressed because he didn’t want to part with the chicken he loved”. (KII, Journalist)
Food diversity	Limited food variety and hidden hunger	“Poor mothers and their children can only fill their stomachs with potatoes, peas, and wheat. A diverse and nutritious diet is also crucial”. (KII, Nutrition expert from the community)
	All is good for the poor	“Only the names of desirable foods can be mentioned, but they cannot be eaten. For the poor and hungry, anything that is available and accessible is acceptable”. (IDI, Mother, 33)
Reliability of food and governance	Commercialization of low-quality junk food	“In the past, people were healthier and happier, free from many diseases. Now, everything is becoming expensive and of poor quality due to a lack of regulation. Milk, medicine, cooking oil—everything is substandard, and there is no one to enforce price and quality controls”. (IDI, Local traditional pharmacist)

Source: Field data.

**Table 8:** Experiences with Nutrition Programs, Policies, and Access.

Themes	Sub-Themes	Narratives
Global impact of private sector and formula milk companies on countries	Formula milk companies hunt for clients in healthcare settings	“Multinational formula milk representatives are allowed to operate in healthcare centers and promote formula milk to parents of malnourished children. After children recover from SAM with the use of formula 75 or 100 and then Ready-to-Use Therapeutic Food (RUTF), mothers are encouraged by doctors and these representatives to continue using their products”. (KII, Nutrition Stabilization Center staff)
	Formula milk companies ‘control over the government	“The deliberate lack of oversight or restrictions on the free movement of formula milk company representatives in hospitals indicates a strong influence of these companies over government institutions and bureaucracy”. (KII)
	Baby food industry advertisement	“The baby food industry frequently misleads and deceives parents about their products. They use labeling to enhance their messaging and boost sales, but restrictions are seldom imposed”. (KII)
	Pakistan Medical Association promotes MNCs	“On what basis is the Pakistan Medical Association running advertisements against open milk? Is it driven by public concern or the funding from multinational companies (MNCs)? Poor farmers sell cow or buffalo milk to these companies at low rates (50–60 rupees), which is then processed into products. In the village, we used to consume open milk and everyone was healthy. The government should investigate these ads and uncover the hidden interests behind them, with the support of the Punjab Food Authority, to ensure transparency and ease in the delivery of open milk”. (KII, journalist)
	Formula milk companies in alliance with the medical community	“Although legislation exists to restrict formula milk, companies bribe medical doctors to promote their products. As of now, a federal board and provincial sub-committees to oversee this issue have not yet been established”. (KII, Health Official)
Barriers to nutrition-specific and sensitive programs	Lack of a sustainable nutrition policy	Historically, the country has lacked a consistent nutrition policy. Policies have frequently shifted, ranging from food distribution and card-based rationing to cash transfers like BISP, and programs such as Safe Motherhood, CMAM, EPI, MNCH, School Health and Nutrition Program, Tawana Pakistan Project, Sasti Roti Scheme, and the recent “No One Sleeps Hungry” initiative. Each government introduces its policies and programs, highlighting the need for a sustainable and consistent approach”. (KII, Nutrition expert)
	Social exclusion of people with low social capital and bureaucratic red-tapism	“Poor and low-caste women often face challenges accessing health and therapeutic programs, while those who are better-off benefit more easily due to their connections with staff and influential figures. To become beneficiaries of the BISP cash program, some women who were missing documentation went to file a complaint but were stopped by the police at the gate. Those who managed to enter the office were shuffled from one department to another, with staff telling them, ‘I can’t help you; go talk to someone else’ or ‘I don’t have time, come back next month.’ The process is exhausting and frustrating, with the poor having to navigate bureaucratic hurdles for years, while the wealthy can get assistance in just minutes”. (IDI, Widow enrolled in BISP Program)
	Sociocultural factors, inadequate care, maternal illiteracy, high fertility, and time poverty	“Poverty, traditional gender roles, social stigma against contraception, preference for male children, and side effects of modern contraceptives are key factors contributing to high fertility rates. Frequent pregnancies and inadequate healthcare lead to maternal malnutrition. The demands of economic activities, caring for the husband and his family, domestic chores, and working in agricultural fields significantly burden mothers”. (KII, Population Officer)
	Inadequate funding deprioritizes nutrition by health bureaucracy	“The CMAM program has become less effective as a significant portion of funds are diverted to other public programs, such as the polio eradication initiative. The coverage of nutrition-related projects is limited due to insufficient budgetary allocations”. (KII, Nutrition Coordinator)
	Insufficient allocation of resources and a shortage of healthcare staff in remote areas	“In South Punjab, a marginalized and underdeveloped region with low literacy rates, structural issues hinder female health workers from filling their designated roles in remote health units. In Southern Punjab, less than half of the Basic Health Units have successfully appointed Lady Health Workers (LHWs) to fill vacancies. For instance, the Rajanpur District Health Information System reported that out of 900 LHW positions, only 650 were filled, leaving 250 positions still vacant”. (KII, Health Official)
	Absenteeism and engaging health workers in non-nutrition programs	“In several remote areas, LHWs are frequently absent. Their excessive involvement in other tasks has led to the deprioritization of nutrition activities within the health department. The workload for LHWs should be reduced, and maternal-child health and nutrition should be given a higher priority on their agenda”. (KII, Healthcare Provider)
	Geographical constraints	“Nutritional aid delivery is frequently limited due to logistical challenges faced by rural and marginalized communities”.
	Other stakeholders’ performance	“Many female school teachers and NGO staff were involved in misusing and selling food that was intended for distribution among girls in rural public schools”. (IDI, Mother, 40)
	Left against medical advice (LAMA) cases	“Most cases of SAM were from poor, geographically isolated, and flood-affected areas. Children with SAM were admitted to the Nutrition Stabilization Center for treatment with antibiotics and formula milk 75 or 100 until they recovered. Poor mothers, fathers, or grandmothers often had to stay at the center to care for their severely ill and malnourished children. However, many of them eventually abandoned the treatment because they needed to care for other children at home”. (KII, Nutrition stabilization center staff)
	Weak system of data management, monitoring, corruption,	“The system for collecting, monitoring, and evaluating data is weak, making strategic planning difficult. Corruption and unethical sales of therapeutic food require monitoring and fair distribution. These issues hinder the effective implementation of nutrition programs”. (KII, Senior Health Official)

Source: Field Data.

## Data Availability

The original contributions presented in the study are included in the article, further inquiries can be directed to the corresponding authors.
